# Lower Postprandial Thermogenic Response to an Unprocessed Whole Food Meal Compared to an Iso-Energetic/Macronutrient Meal Replacement in Young Women: A Single-Blind Randomized Cross-Over Trial

**DOI:** 10.3390/nu12082469

**Published:** 2020-08-17

**Authors:** Alex E. Mohr, Carmen Ramos, Kelvin Tavarez, Paul J. Arciero

**Affiliations:** 1 College of Health Solutions, Arizona State University, Phoenix, AZ 85004, USA; aemohr@asu.edu; 2 Department of Health and Human Physiological Sciences, Skidmore College, Saratoga Springs, NY 12866, USA; cramos@skidmore.edu (C.R.); ktavarez@skidmore.edu (K.T.); 3 Human Nutrition and Metabolism Laboratory, Skidmore College, Saratoga Springs, NY 12866, USA

**Keywords:** meal replacement, nutritionally engineered food, ultra-processed food

## Abstract

In contrast to ultra-processed foods that are associated with increased weight gain and obesity risk, nutritionally engineered dietary supplements, including meal replacement (MR) bars and shakes, are generally promoted as healthy. Limited data is available comparing the metabolic and hunger responses of whole food (WF) versus MR meals. The purpose of this study was to directly compare the thermic effect (TEM), respiratory exchange ratio (RER), hunger/taste ratings, and glucose response of two different breakfast meals containing MR and WF products in young healthy women. Eight volunteers completed two iso-caloric (529 kcals)/macronutrient (50% carbohydrates; 26% fat; 24% protein) test meals in a single-blind, randomized crossover design: (1) whole food meal; or (2) meal replacement. TEM was significantly higher following MR compared with WF (percent mean difference: 7.76 ± 3.78%; absolute mean difference: 0.053 ± 0.026 kcal/minute, *p* = 0.048), whereas WF substrate utilization demonstrated lower carbohydrate oxidation (RER) than MR (mean difference: −0.024 ± 0.008, *p* = 0.005). No differences existed for blood glucose response and feelings of hunger, desire to eat, and satiety among trials. Consumption of an MR meal increases postprandial thermogenesis and RER compared to a WF meal, which may impact weight control and obesity risk over the long-term.

## 1. Introduction

The demand and consumption of ultra-processed/refined food (PF) options, including packaged, pre-made, and fast food items, have steadily increased over recent decades and are becoming a dominant feature in the global food system [1,2]. Unfortunately, obesity and metabolic disorders are often associated with increased consumption of ultra PFs [3,4,5]. Paralleling this increased consumption of ultra PF is the increased intake of dietary supplements [6], including meal replacement (MR) bars and shakes, which unlike other PFs, are generally promoted as healthy. Indeed, MR options are often used in weight loss interventions due to similar or better nutritional values compared to whole food (WF) mixed meals [7,8]. As such, these ‘nutritionally engineered’ MR foods exhibit certain superior health benefits compared to many WF options. Specifically, medium to long-term weight loss interventions using nutritionally engineered MR shakes and/or bars have demonstrated enhanced weight/fat loss, improved cardiometabolic outcomes, and dietary adherence compared to traditional WF meal options [9,10,11,12]. However, it is less understood whether these products may increase postprandial thermogenesis and decrease hunger responses to an acute meal challenge in younger healthy adults.

Previous work from our laboratory has assessed both acute and chronic effects of different meal compositions and ingestion patterns utilizing nutritionally engineered MR meals [12,13,14]. For instance, compared to a traditional feeding schedule of three meals per day, increasing protein intake evenly throughout the day (protein pacing) improved body composition and metabolism over an eight-week intervention [12]. Further, a pancake meal breakfast combining resistant starch and whey protein was found to increase fat oxidation, peptide YY, and satiety response compared to a carbohydrate starch control, resistant starch alone, and control starch with protein [13]. More recently, we have examined the acute effects of sandwich meals with differing levels of food processing on postprandial thermogenesis, substrate utilization, hunger/taste ratings, and glucose response [14] and reported a PF meal consisting of gluten free (GF) ingredients produces a significantly lower thermogenic response (calorie burn) compared to a WF or ultra PF meal of equal caloric and macronutrient composition. In addition, the WF meal resulted in a greater sensation of fullness compared to GF and PF conditions. This line of research has substantial public health implications regarding how specific dietary advice is recommended based on food processing and ingredient selection to better optimize body weight control and composition.

Another important consideration is the physical form of the test meals, with multiple studies reporting greater energy intake and appetite after acute meal challenges with liquid compared to solid food forms [15,16,17], although this is not a universal finding [18,19,20]. Many of the food products used in these studies were single items, and therefore, less reported is the effect of MR liquid shakes and/or bars in comparison to a mixed, WF meal. Previous research in older adults and adolescents shows acute ingestion of a breakfast MR blunts satiety and increases subsequent food intake [21,22,23,24,25]. To our knowledge, only one study has directly compared a breakfast MR to a WF cereal and milk meal in young adults, showing no differences in thirst, hunger, appetite, or subsequent dietary intake [26]. Less data exists on the thermogenic responses to acute MR versus WF meal challenges in young healthy women. Given the adverse body weight and composition changes during the transition from high school to university living [27], college-aged women are susceptible to weight gain due to poor nutrition habits [28]. Based on this previous literature, it was hypothesized that energy expenditure and satiety would be greater in the WF compared to MR breakfast meal, whereas glucose and substrate utilization would be similar.

Thus, a better understanding of the metabolic and hunger responses to MR versus WF meals may provide important information regarding improved body weight management and obesity risk in young adult women. Therefore, the purpose of this study was to systematically compare the thermogenic response, hunger ratings, and blood glucose of MR and WF breakfast meals matched for energy and macronutrient composition in healthy, young college-aged women.

## 2. Materials and Methods

### 2.1. Participants

A total of 10 women from the Saratoga Springs, NY local college (Skidmore College) were recruited through email and initially screened, of whom all were eligible for participation during the spring of 2015. Participants were non-smoking, healthy women between the ages of 18–24 years with no known cardiovascular or metabolic diseases as assessed by a medical history and examination by their personal physicians. Participants with lactose intolerance/sensitivity, diabetes mellitus, and hypoglycemia were ineligible to participate in the study. To prevent possible influence on data, participants who engaged in excess use of dietary supplements were also excluded from the study. Each participant provided informed written consent in adherence with the Skidmore College Human Subjects review board prior to participation, and the study was approved by the Human Subjects Institutional Review Board of Skidmore College (IRB#: 1302-333). All experimental procedures were performed in accordance with the Federal Wide Assurance and related New York State regulations, which are consistent with the National Commission for the Protection of Human Subjects of Biomedical and Behavioral Research and in agreement with the Helsinki Declaration as revised in 1983. This trial was registered at clinicaltrials.gov as NCT04453254.

### 2.2. Experimental Design

Upon recruitment, participants visited the Human Nutrition and Metabolism Laboratory for a 1 h initial meeting to complete a medical questionnaire and informed consent form as well as have their body composition assessed via a dual-energy x-ray absorptiometry (DXA) scan. Measurements of height and body weight were taken using standard laboratory equipment and procedures. Pre-testing instructions were also given, which consisted of (a) refraining from eating or drinking 8–10 h before testing, (b) eating and drinking normally for the 48 h preceding the 8–10 h fast, (c) refraining from drinking coffee or alcohol 48 h prior to testing, (d) preventing any excess physical activity the day prior to testing, and (e) maintaining a food log for the three preceding days of testing.

**Figure 1:**
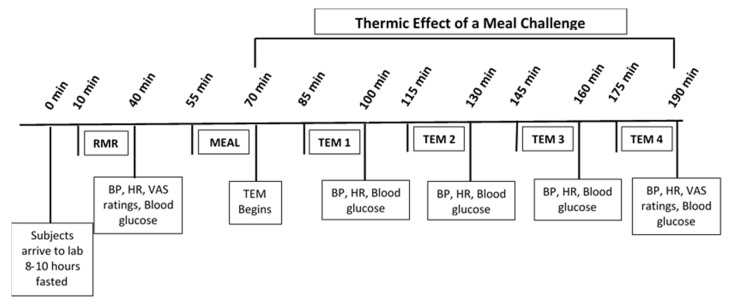
Timeline of thermic effect of a meal (TEM) test days. RMR: resting metabolic rate; BP: blood pressure; HR: heart rate; VAS: visual analog scales for hunger, fullness, satiation, desire to eat.

Participants were instructed to go to bed by 11 pm the night prior to testing and were transported by car to the Human Nutrition and Metabolism Laboratory by 6:30 am. Upon arriving to the lab, baseline measurements were conducted and participants were then given 15 min to ingest the assigned meal. Subsequent measurements were conducted as outlined in Figure 1.

After a one-week washout period, the participants returned to the lab to repeat the same procedure with the other test meal. The order in which the meals were prescribed was random and balanced.

### 2.3. Test Meals

On two separate visits to the Human Nutrition and Metabolism Laboratory, all subjects consumed one of two test meals in a single-blind, randomized repeated-measures crossover design: (1) meal replacement meal (MR; *n* = 8); (2) whole foods meal (WF; *n* = 8). The WF meal and the MR meal were composed so that caloric and macronutrient composition were equal Table 1.

### 2.4. Resting Metabolic Rate (RMR) and Thermic Effect of a Meal (TEM)

RMR was measured upon arrival using the ventilated hood system with a computerized open-circuit indirect calorimeter as previously described [13]. Briefly, upon arrival to the lab, participants rested quietly for 10 min and then were instructed to lie down and have their resting metabolic rate (RMR) measured for 30 min using a metabolic cart, ventilated hood system, and mixing chamber (ParvoMedics TrueOne 2400 Metabolic Measurement System, Sandy, UT, USA). Following RMR and other baseline measurements, participants underwent a thermic effect of a meal (TEM) challenge within 15 min. Following meal ingestion, participants rested quietly for 2 h during which time their RMR was measured every other 15 min for 15 min (i.e., measurements taken at minutes: 15–30, 45–60, 75–90, and 105–120, postprandially).

### 2.5. Respiratory Exchange Ratio (RER)

RER was calculated from indirect calorimetry gas exchange as the volume of carbon dioxide produced to the volume of oxygen consumed (VCO_2_/VO^2^) (Parvomedics, Truemax 2400, Sandy, UT, USA) and were calculated using the exact same method described above for 120 min TEM.

### 2.6. Visual Analog Scale (VAS) Measures

Baseline measurements of subjective ratings of hunger, fullness, and desire to eat were evaluated using a 100 mm visual analog scale (VAS). Participants were prompted with four questions regarding hunger, fullness, and desire to eat and were then asked to draw a vertical line on the VAS scale representing how they felt at that moment. A vertical line mark at 0 mm represented no feelings, whereas a mark at 100 mm indicated extreme feelings. The degree to which each sensation was felt was quantified by measuring how far the mark was from the 0 mm point. A standard millimeter ruler was used for all measurements and all scores were computed by the same investigator. At the completion of the 120 min test period, hunger, fullness, and desire to eat were all reevaluated.

### 2.7. Blood Pressure and Heart Rate

Resting heart rate and blood pressure were manually recorded in the supine position as previously described [13]. Heart rate was obtained by palpation from the radial pulse for 1 min and blood pressure measurements were obtained using a sphygmomanometer (American Diagnostic Corp., 55 Commerce Drive, Hauppauge, NY, USA) and stethoscope (Littman Quality, USA, St. Paul, MN, USA) by a trained investigator on each of the three test meal days following the RMR measurement.

### 2.8. Blood Glucose Measure

Immediately following the RMR measurements, subjects underwent a finger stick for the assessment of blood glucose using a OneTouch Blood Glucose Analyzer (LifeScan IP Holdings, LLC., Malvern, PA, USA). Blood glucose were then measured every 30 min thereafter.

### 2.9. Data Analysis

Normality statistics (Shapiro–Wilk’s tests and skewness and kurtosis z-scores) and probability plots (Q-Q plots and histograms) were generated to test normality assumptions, and log transformations were performed as appropriate. Area under the curve (AUC), and absolute and percent changes were conducted as described previously [13,29]. Briefly, blood glucose and thermic effect of meal (TEM) area under curve (AUC) were calculated by the trapezoidal rule for the entire 120 min postprandial period as an additional evaluative method. TEM of each meal was calculated using the difference between baseline energy expenditure levels and the increase in energy expenditure throughout the two hours of testing post-consumption of the meal. To determine the effect of meal type on the outcome variables, linear mixed-effects models were employed with a random intercept for subject, and time and meal type as fixed factors. Additionally, to assess whether the effect of meal type was different between time points, an interaction term was added to the model (meal*time). This model provides unbiased estimates of time and treatment effects under a missing-at-random assumption. Relevant covariates (i.e., body mass index (BMI), age, and baseline values) were added to the model to adjust for possible confounding. Multiple comparisons were made on generated estimated marginal means for both main effects (time and meal) and interaction effects (time*meal) with Bonferroni post-hoc tests. Absolute postprandial TEM and glucose AUC were compared by independent *t*-tests. Due to the limited data in this area, power analysis and sample size were based on previous research comparing the thermic effect of breakfast meals with differing macronutrient composition in healthy young females [28]. Based on an estimated effect size of *d* = 1.60, with a power = 0.80 and α-level at 0.05, we estimated a total sample size of eight participants to detect a significant effect in postprandial TEM between meals (G*Power 3.1). All analyses were performed using SPSS 26.0 for Windows (SPSS Inc.). An α-level was set at a significance of *p* < 0.05. Data are shown as mean values (with standard error) unless otherwise noted.

## 3. Results

### 3.1. Participants and Compliance

The physical characteristics of the eight college-aged females in the study are presented in Table 2.

The nutrient composition of the day preceding testing consisted of a mean 1493 ± 361 kcal, 46.5 ± 14.7% of calories from carbohydrates, 20.1 ± 5.6% from protein, and 33.4 ± 11.0% from fat (Table 3).

### 3.2. Resting Metabolic Rate (RMR) and Thermic Effect of a Meal (TEM)

**Figure 2:**
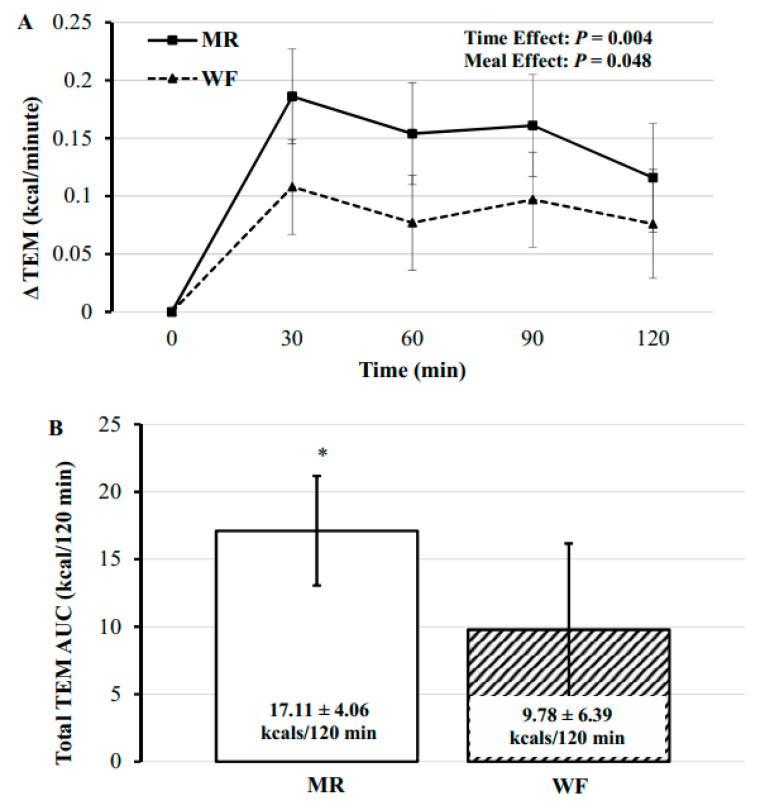
(**A**) Postprandial change in TEM across 120 min for each meal. (**B**) Effect of test meals on the 120 min postprandial period area under the curve (AUC) for thermic effect of meal following the two test meals. MR: meal replacement meal; WF: whole food meal. Data displayed as mean ± standard error. * MR significantly higher TEM than WF; *p* < 0.05.

There were significant main effects of time and meal type on TEM (*F*(4, 66.04) = 4.25, *p* = 0.004 and *F*(1, 65.98) = 4.06, *p* = 0.048, respectively; Figure 2A,B).

Postprandial TEM values were significantly increased from baseline at all time points (*p* ≤ 0.015). In addition, TEM was significantly greater for MR compared to WF (percent mean difference: 7.76 ± 3.78%; absolute mean difference: 0.053 ± 0.026 kcal/minute, *p* = 0.048), with no significant differences at individual time points between meals (*F*(4, 65.89) = 0.35, *p* = 0.845; Table 4).

### 3.3. Respiratory Exchange Ratio

**Figure 3:**
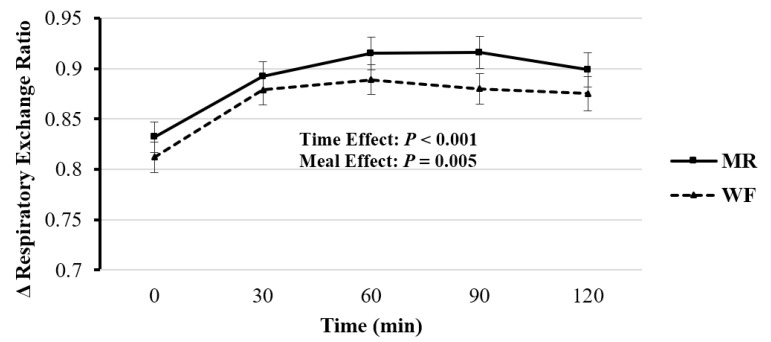
Postprandial change in respiratory exchange ratio 120 min for each meal. MR: Meal replacement meal; WF: Whole food meal. Data displayed as mean ± standard error.

There was a significant main effect of time for substrate utilization as measured by indirect calorimetry (RER; *F*(4, 66.15) = 13.17, *p* < 0.001) and meal, (*F*(1, 65.99) = 8.31, *p* = 0.005; Figure 3; Table 4). Overall, WF had a significantly lower RER value compared to MR (mean difference: −0.024 ± 0.008, *p* = 0.005). Similar to TEM, there were no significant differences at individual time points between meal types (*F*(4, 65.81) = 0.208, *p* = 0.933).

### 3.4. Blood Glucose

**Figure 4:**
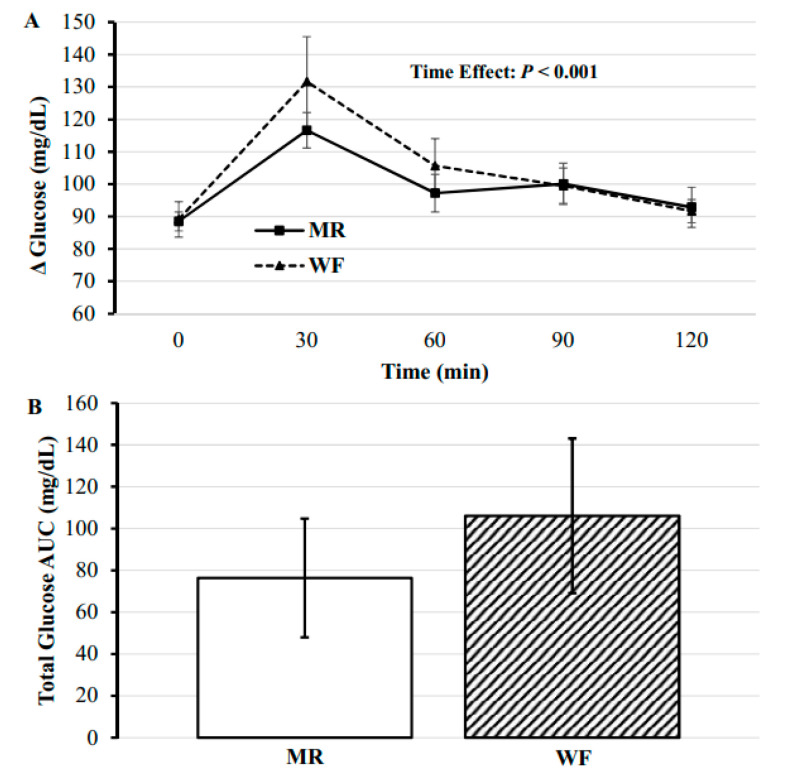
(**A**) Postprandial change in glucose across 120 min for each meal. (**B**) Effect of test meals on the 120 min postprandial period area under the curve for glucose of meal following the two test meals. MR: meal replacement meal; WF: whole food meal. Data displayed as mean ± standard error.

The main effect of time was significant, (*F*(4, 65.25) = 16.46, *p* < 0.001), with significant changes in blood glucose concentrations occurring at 30, 60, and 90 min compared to baseline (mean Δ: 10.98–35.31 mg/dL, *p*s ≤ 0.029; Figure 4A; Table 5). However, the effects of meal type and meal*time were not significant, (*F*(1, 65.17) = 1.88, *p* = 0.175 and *F*(4, 64.92) = 1.07, *p* = 0.379, respectively). Furthermore, AUC for blood glucose did not differ significantly between the two test meals, (*T*(14) = −638, *p* = 0.534; Figure 4B).

### 3.5. Feelings of Hunger, Desire to Eat, and Fullness

Pre- and post-values for the subjective ratings of hunger, desire to eat, and fullness are presented in Table 6. Both hunger and desire to eat significantly decreased after consumption of the test meals, (*F*(1, 24) = 26.49, *p* < 0.001 and *F*(1, 24) = 17.37, *p* < 0.001, respectively). However, there were no significant differences for hunger and desire to eat for the effect of meal (*F*(1, 24) = 0.63, *p* = 0.44 and *F*(1, 24) = 2.06, *p* = 0.16, respectively) or meal*time (*F*(1, 24) = 1.14, *p* = 0.29 and *F*(1, 24) = 1.51, *p* = 0.23, respectively). Fullness was significantly increased after consumption of both meals, (*F*(1, 24) = 29.85, *p* < 0.001), but similar to hunger and desire to eat, there were no significant differences between meals, although a strong trend existed (*F*(1, 24) = 0.19, *p* = 0.66, or meal*time, *F*(1, 24) = 3.48, *p* = 0.07).

## 4. Discussion

The primary aim of this study was to compare a WF to a MR meal matched for energy and macronutrient content on the thermic response of a meal (TEM), respiratory exchange ratio (RER), blood glucose, and fullness, hunger, and satiety ratings. The main findings of the current study reveal that: (1) a MR meal elicited a significantly larger thermogenic response and (2) had a higher RER (greater carbohydrate oxidation) compared to the WF meal. However, blood glucose and subjective ratings of fullness, hunger, and satiety did not differ significantly between the two meals. Taken together, the current findings demonstrate an acute MR meal challenge produces a significantly higher thermogenic response (calories burned) and higher carbohydrate oxidation compared to a WF meal of equal caloric and macronutrient composition. Further, a MR meal is comparable in blood glucose response and sensation of fullness, hunger, and satiety compared to a whole food meal. These findings may have important implications regarding how specific dietary advice is recommended based on incorporating MR for both weight control, especially among groups at higher risk of weight gain/obesity, such as younger adults in university/college settings.

### 4.1. Thermic Effect of a Meal (TEM)

The main findings of the current study indicate there was a significant main effect in thermogenesis following consumption of the test meals, with an MR meal displaying an increased thermogenic response compared to a WF meal. Our lab has previously demonstrated a significantly greater TEM response (67–100%) following eight weeks of a higher protein intake (34% of total calories) and frequency (six meals/day), known as ‘protein pacing’, with the support of three nutritionally engineered MR meals of liquid shakes and bars compared to three traditional unrefined/unprocessed WF meals/day [12]. The increased TEM was associated with significant reductions in total and abdominal body fat and increased lean body mass. More recent work from our laboratory has shown a gluten-free (GF) meal reduced TEM compared to a WF meal and an ultra PF meal [14]. To our knowledge, the current study is the first direct comparison of an acute meal challenge of iso-caloric/macronutrient meals containing nutritionally engineered MR foods (shakes and bars) versus unrefined WF. Recent data by Hall and colleagues [30] highlighted the ability of a diet composed of ultra-PF to promote increased energy intake (via increased carbohydrate and fat consumption) and weight gain compared to an unprocessed WF diet matched for calories, energy density, macronutrients, sugar, sodium, and fiber. Ultra PF have been defined as, “formulations mostly of cheap industrial sources of dietary energy and nutrients plus additives, using a series of processes” and containing minimal whole foods [31]. While the MR condition in the present study may be considered processed, much of the ingredients are of generally high nutritional values, including 32 g (24% of total kcal) of protein. Further, much of the meal replacement shakes and bars available to consumers have been ‘nutritionally engineered’ for improved health outcomes compared to ultra PF formulated for increased appetitive properties and often high in calories, salt, sugar, and fat [32,33,34]. According to the ‘protein leverage hypothesis’ proposed by Simpson and Raudenheimer [35], a reduced protein density diet may increase intake of ultra PF and drive energy overconsumption and obesity, a finding supported by some [36], but not all investigators [30]. In support of our findings, recent data comparing processed MR formulations from either diary or plant sources showed significantly higher TEM in comparison to carbohydrate controls in young healthy volunteers [37,38].

In the current study, energy and macronutrient composition were well-matched with both meal formulations, including identical protein amounts (32 g), although sugar (WF: 27.6 g vs. MR: 50.7 g) and fiber content (WF: 19.7 g vs. MR: 1.7 g), were different. In contrast to our results, an acute crossover meal challenge using an iso-caloric (600 kcal) sugar only beverage vs. a mixed-nutrient beverage (17% protein; 67% carbohydrate; 17% fat) found TEM to be significantly lower in the sugar only beverage [39]. While the meal formulations in the current study focused on WF versus MR, future studies should explore the effects of varying amounts and types of sugar, including glycemic index and load, on TEM. In addition to sugar, the difference in fiber content (and type) may also affect TEM on and acute and longer-term basis using WF and MR interventions. Specifically, research from our laboratory has shown an acute meal challenge of protein combined with resistant starch fiber (RS4) significantly increases substrate oxidation compared to no fiber control meal. Thus, future investigations should explore fiber content and type and its potential impact on TEM, as well as the gut microbiota and microbially-derived products (e.g., short-chain fatty acids). Another important factor that may have influenced our findings was the potential difference in digestion rates of the two meals. While tube-delivered meals have been reported to not differ in magnitude or duration of TEM after orally ingested meals [40], future research should more accurately compare the solid vs. liquid formats of meals. Lastly, it’s possible other components not controlled for in the current study, such as micronutrients, may have contributed to the change in TEM.

TEM represents the energy expenditure of processing and storing food, as well as the metabolic effects of the influx of nutrients. It may be possible to alter TEM as a weight-loss tool in both research and clinical settings, however the current body of literature remains very limited [41]. The findings of our study provide some of the first evidence that a nutritionally engineered MR may offer slightly enhanced postprandial thermogenesis (energy expenditure) compared to an iso-caloric/macronutrient WF breakfast. Over time, this increase in TEM may result in significant weight loss.

### 4.2. Respiratory Exchange Ratio (RER)

While both meals contained the same amount of carbohydrate (69 g) and fat (16 g), the MR meal showed an overall greater RER value compared to the whole food meal. As with TEM, to our knowledge, no study has directly compared the effects of these two iso-caloric, macronutrient matched meal formats on RER. It is likely that greater sugar content in the MR may have partially been responsible for these findings. Indeed, this finding aligns with a number of previous acute randomized cross-over trials investigating energy expenditure, substrate utilization, and RER [42]. For example, iso-caloric mixed-meal challenges using higher vs. lower glycemic index and glycemic load meals also elicited greater RER levels [43]. While glycemic index/load has been a nutrition topic of interest for decades, matching meals for protein and macronutrients presents a fascinating new area of research with important energy balance implications [44].

### 4.3. Blood Glucose Response

The current study showed no differences among the two test meals on blood glucose response, suggesting nutrient composition and meal form, including the degree of processing, had little influence on blood glucose. This finding corroborates our previous research and others showing acute meal ingestion shows similar blood glucose responses to varying processed meals [13,45,46], though, there is research that conflicts with these findings [47]. In contrast to the current study, most previous research reporting lower glucose responses to meal ingestion, used test meals with varying amounts of fiber, protein, or fat [48]. While the macronutrient compositions of the present test meals were nearly identical, the difference in meal form and sugar amount were notable differences between the two meals. Indeed, there is strong agreement that ultra-processing disrupts the physical structure of intact food, making the macromolecular structure more simple and thus easier and faster to digest, resulting in more rapid increases in plasma glucose and insulin [49]. While insulin was not measured, the current study does not support that a nutritionally engineered meal adversely affects glycemic control compared to a whole food meal, at least in young healthy, insulin-sensitive women.

### 4.4. Feelings of Satiety, Hunger, Desire to Eat

As previously noted, there was no significance found between the two meals in regards to desire to eat, satiety, and hunger. In relation to the difference noted in the thermogenic response between the two test meals, TEM has previously been suggested as one of the mechanisms that influences appetite sensations, including satiety [50]. Further, acute meal challenges comparing high-sugar to mixed-nutrient beverages have reported significantly lower levels of satiety [39]. However, in a more recent meta-analysis no associations were found between change in TEM and various appetite and satiety measures [51]. Findings from the present study, while null, are nonetheless intriguing and novel as a processed, nutritionally engineered MR had similar hunger, satiety, and fullness ratings compared to a high-quality, unprocessed WF meal. While future research should assess these measures on additional acute time points (instead of pre/post), these findings were not surprising considering the matched energy and macronutrient compositions of the two meals.

### 4.5. Limitations

There are several limitations to the current study. The relatively small sample size, age range, and ethnicities of participants limits the extrapolation of our results. However, due to the use of a randomized cross-over design, the results of this research are compelling and warrant further investigation with stronger powered sample sizes with participants of varying ages and ethnicities. Another limitation was the VAS questionnaires were administered pre/post-testing and therefore more time-sensitive changes in response may have been missed. Finally, this acute study spanned a time frame of 120 min. Therefore, TEM measures beyond 2 h may provide further information and insight regarding the differences between a WF and MR meal. It’s important to note, previous studies highlight the major part of TEM takes place during the first few hours after ingestion of a meal [52,53].

## 5. Conclusions

In conclusion, our novel findings suggest that a processed, nutritionally engineered MR increases postprandial thermogenesis and RER compared to a high-quality, unprocessed WF meal of equal caloric and macronutrient composition. Importantly, we found no difference in the postprandial blood glucose response or subjective ratings of satiety, hunger, and desire to eat between the two meals, highlighting the similarity of these meals. While a diet composed of ultra PF, yet similar total calories and low in nutrient value may promote weight gain and obesity, we show for the first time in an acute meal challenge that a processed, nutritionally engineered MR meal may offer an effective strategy for obesity prevention and treatment.

## Figures and Tables

**Table 1:** Nutritional analysis and ingredients of the two test meals.

Nutritional Analysis	WF	MR
Energy (kcal)	529	529
Total carbohydrate (g)	69 (50%)	69 (50%)
Total sugar (g)	27.6	50.7
Total fat (g)	16 (26%)	16 (26%)
Total saturated fat (g)	5.3	3.8
Total protein (g)	32 (24%)	32 (24%)
Total fiber (g)	19.7	1.7
**Ingredients**	2% milk (305 g)	Boost High-Protein Nutritional Drink (224 g)
	Kashi Go-Lean Original cereal (63 g)	Canola oil (3 g)
	Almonds (28 g)	Honey (21 g)
	Raspberries (41 g)	Met-Rx Big 100 Colossal Crispy Apple Pie Meal Bar (63 g)
	Strawberries (28 g)	

WF: whole food meal; MR: meal replacement.

**Table 2:** Subject characteristics ^a^.

*N*	8
Age (years)	20.37 ± 0.92
Weight (kg)	69.18 ± 14.51
Height (cm)	168.76 ± 5.73
BMI	24.17 ± 4.26
Ethnicity/Race	
Caucasian	1 (12.5%)
Hispanic	5 (62.5%)
Black/African American	1 (12.5%)
Other	1 (12.5%)
Percent fat mass (%)	32.80 ± 8.28
Systolic BP (mmHg)	106.00 ± 12.38
Diastolic BP (mmHg)	60.25 ± 7.59
Resting HR (bpm)	63.25 ± 15.37

^a^ Data presented as mean ± standard deviation or *N* (%). BMI, body mass index; HR, heart rate; BP, blood pressure.

**Table 3:** Dietary nutrient composition the day preceding testing ^a^.

Nutrient	
Calories (kcal)	1493.99 ± 361.37
Carbohydrate (%)	46.50 ± 14.71
Protein (%)	20.09 ± 5.57
Fat (%)	33.41 ± 10.96
Saturated Fat (g)	17.87 ± 7.19
Monounsaturated Fat (g)	7.71 ± 5.36
Sugar (g)	54.03 ± 38.14
Fiber (g)	18.97 ± 9.93
Iron (mg)	14.34 ± 6.93
Sodium (mg)	2692.55 ± 1038.98
Potassium (mg)	1022.94 ± 490.46
Omega 3 Fatty Acid (g)	0.27 ± 0.17
Omega 6 Fatty Acid (g)	1.41 ± 0.71
Caffeine (mg)	7.75 ± 21.92

^a^ Data presented as mean ± standard deviation.

**Table 4:** RMR, TEM, and RER during the test meals ^a^.

Outcome Variable	Meal	Baseline	30 min TEM	60 min TEM	90 min TEM	120 min TEM
RMR ^b^/TEM ^c^	MR *	0.93 ± 0.06	3.42 ± 1.83	2.78 ± 1.70	7.73 ± 1.17	6.59 ± 1.71
	WF	0.94 ± 0.06	5.93 ± 1.71	1.62 ± 1.17	4.39 ± 1.70	3.76 ± 1.68
RER ^d^	MR *	0.83 ± 0.01	0.89 ± 0.02	0.92 ± 0.02	0.92 ± 0.01	0.89 ± 0.01
	WF	0.81 ± 0.01	0.87 ± 0.03	0.87 ± 0.02	0.86 ± 0.01	0.86 ± 0.01

^a^ Effects based on estimated marginal means, actual values displayed (mean ± standard error). ^b^ RMR: resting metabolic rate, kcal/minute. ^c^ TEM: Thermic effect of meal values calculated as incremental area under the curve for 0–30, 30–60, 60–90, and 90–120-min periods, postprandially. ^d^ RER: respiratory exchange ratio. MR: meal replacement meal; WF: whole food meal. * Significant main effect of meal: MR > WF; *p* < 0.05.

**Table 5:** Effects of meal type on mean blood glucose at baseline and 30, 60, 90, and 120 min in the postprandial period ^a^.

Outcome Variable	Meal	Baseline	30 min	60 min	90 min	120 min
Glucose, mg/dL	MR	88.50 ± 2.92	116.63 ± 5.47	97.22 ± 5.79	100.07 ± 6.38	92.85 ± 6.18
	WF	89.13 ± 2.34	131.63 ± 13.89	105.63 ± 8.41	99.51 ± 5.78	91.68 ± 3.58

^a^ Effects based on estimated marginal means, actual values displayed (means ± standard error). MR: meal replacement meal; WF: whole food meal.

**Table 6:** Effects of meal type on mean values of hunger and satiety rating outcome measurements at baseline and 120 min in the postprandial period ^a^.

Outcome Variable	Meal	Baseline	120 min
Hunger (mm)	MR	52.00 ± 12.27	20.75 ± 7.67
	WF	54.13 ± 13.09	6.50 ± 2.56
Desire to eat (mm)	MR	70.50 ± 10.39	21.63 ± 6.31
	WF	46.37 ± 12.90	19.75 ± 11.82
Feeling of Fullness (mm)	MR	40.63 ± 10.52	67.75 ± 11.00
	WF	29.88 ± 11.40	85.13 ± 4.04

^a^ Effects based on estimated marginal means, actual values displayed (means ± standard error). MR: meal replacement meal; WF: whole food meal.

## Abbreviations

The following abbreviations are used in this manuscript:

DXA	Dual-Energy X-ray Absorptiometry scan
MR	meal replacement meal
WF	whole food
RMR	resting metabolic rate
TEM	thermic effect of a meal
BMI	body mass index
RER	respiratory exchange ratio
AUC	area under the curve
VAS	visual analog scale
